# A Time-Domain CMOS Oscillator-Based Thermostat with Digital Set-Point Programming

**DOI:** 10.3390/s130201679

**Published:** 2013-01-29

**Authors:** Chun-Chi Chen, Shih-Hao Lin

**Affiliations:** Department of Electronic Engineering, National Kaohsiung First University of Science and Technology, Kaohsiung 811, Taiwan; E-Mail: u9652007@nkfust.edu.tw

**Keywords:** CMOS, oscillator, temperature sensor, thermostat, time-domain

## Abstract

This paper presents a time-domain CMOS oscillator-based thermostat with digital set-point programming [without a digital-to-analog converter (DAC) or external resistor] to achieve on-chip thermal management of modern VLSI systems. A time-domain delay-line-based thermostat with multiplexers (MUXs) was used to substantially reduce the power consumption and chip size, and can benefit from the performance enhancement due to the scaling down of fabrication processes. For further cost reduction and accuracy enhancement, this paper proposes a thermostat using two oscillators that are suitable for time-domain curvature compensation instead of longer linear delay lines. The final time comparison was achieved using a time comparator with a built-in custom hysteresis to generate the corresponding temperature alarm and control. The chip size of the circuit was reduced to 0.12 mm^2^ in a 0.35-μm TSMC CMOS process. The thermostat operates from 0 to 90 °C, and achieved a fine resolution better than 0.05 °C and an improved inaccuracy of ± 0.6 °C after two-point calibration for eight packaged chips. The power consumption was 30 μW at a sample rate of 10 samples/s.

## 1. Introduction

The increase in circuit density and clock speed of modern VLSI systems cause chips to run hotter, resulting in thermal problems. The components in these systems can be damaged by temperatures outside their operation ranges if proper protection is not implemented. Because heat cannot be removed quickly, careful thermal management techniques must be incorporated into all modern system designs.

**Figure 1.**
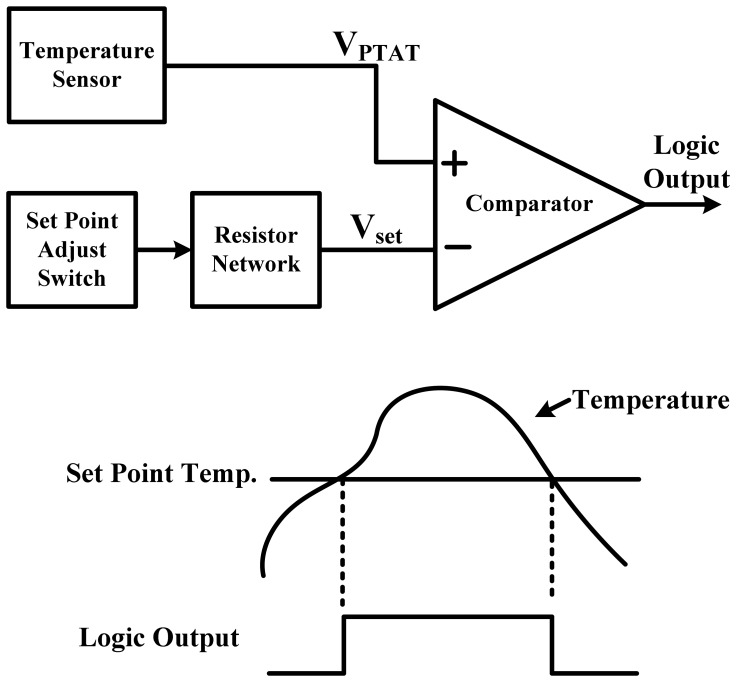
Simplified example of voltage-domain thermostat with logic output.

An on-chip temperature sensor with set-point programming, which functions as a thermostat, is required for temperature monitoring in various applications to prevent overheating or overcooling. They are mounted close to the microprocessor or other crucial heat sources and constantly monitor the operation temperature. Their features include logic-level output to indicate whether the temperature is above or below a preset value and a user-programmable temperature setting. A temperature sensor with a logic output is similar to a voltage output sensor, except that the output amplifier is replaced with a comparator, as shown in Figure 1. Temperature set-point programming is usually accomplished using an external resistor network or DAC [1–4]. The resistor values for achieving the desired trip-point temperatures are calculated using a universal formula relating to resistance. The output is high for V_PTAT_ > V_set_, and can be used to directly control the fan speed in a thermal management system to avoid thermal damage. Conversely, thermostats can also be used to avoid low temperature conditions. However, because the set-point cannot be digitally configured, it is costly to program multiple set-points, which is unsuitable for full VLSI integrations.

**Figure 2.**
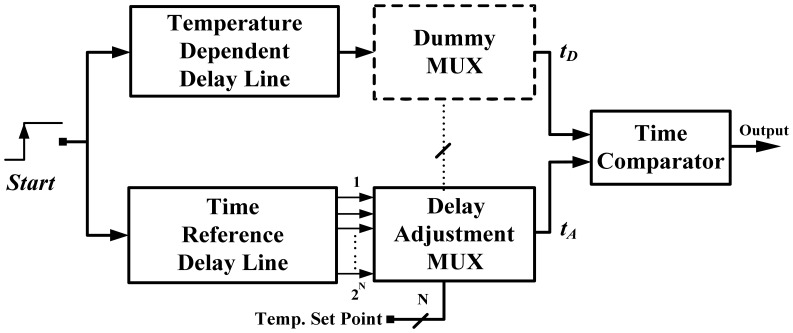
Block diagram of former time-domain thermostat.

A time-domain thermostat substantially reduces the cost and power of temperature sensors for VLSI integration [5], as shown in Figure 2. The temperature dependent delay *t_D_* proportional to absolute temperature (PTAT) is produced by a simple delay line, and the adjusted delay *t_A_* is generated by another thermal-compensation reference delay line with a tap-point set by a multiplexer (MUX). A larger MUX input results in a later stage of the reference delay line being tapped out and a longer adjusted delay *T_A_*. The final set-point comparison was achieved using a simple D-type flip-flop (DFF) as the time comparator. Because all signals are processed in a time-domain instead of a conventional voltage-domain, the performance can be enhanced by scaling down the fabrication process. In addition, the circuit is composed of digital gates without the requirement of a BJT, DAC, or OPAMP, and the sensor can be easily integrated into the VLSI systems. However, the delay-line-based structure consumes a larger area with increasing resolution. With an 8-bit MUX design and more than 256 stages of delay cells with thermal compensation, the resolution was only 0.5 °C and the chip size was 0.4 mm^2^ in a TSMC 0.35-μm CMOS process. Moreover, because a significant curvature occurred on the transfer curve of the thermal delay *t_D_*, the sensor achieved a measurement error of ±1.0 °C over a temperature range of 0–75 °C after two-point calibration. For cost reduction and accuracy enhancement, this paper proposes a thermostat with two oscillators that are suitable for time-domain curvature compensation instead of using linear delay lines. A simple time comparator with a built-in hysteresis can provide an effective temperature alarm and temperature control. The remainder of this paper is organized as follows: Section 2 introduces the detailed circuits of the proposed thermostat; Section 3 presents the experiment results of the circuit; and lastly, Section 4 presents our conclusions.

## 2. Main Architecture

**Figure 3.**
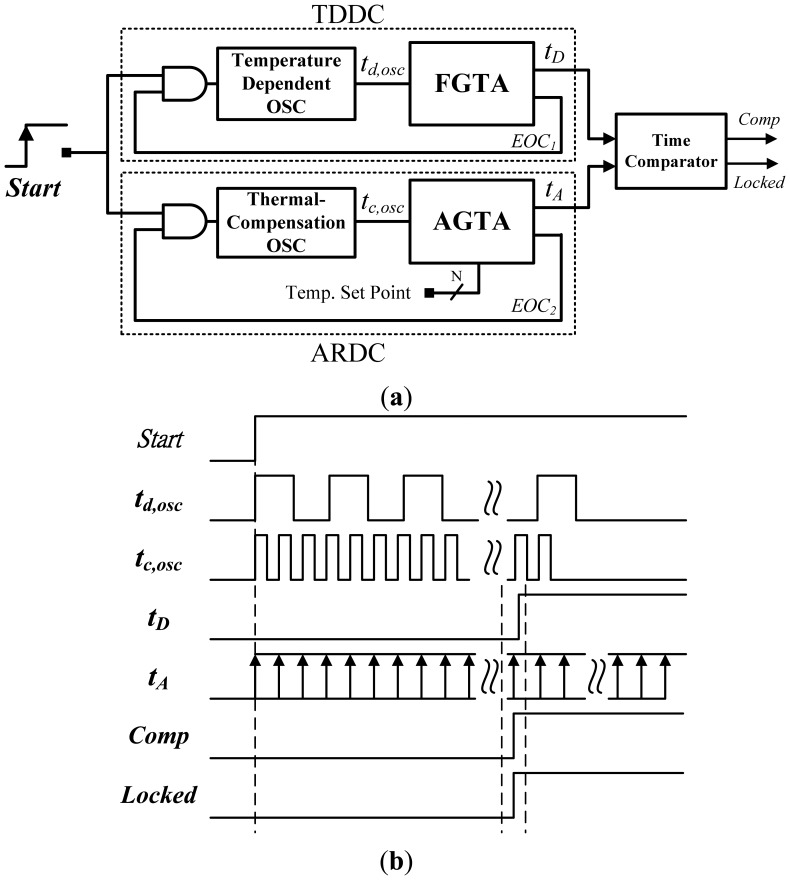
(**a**) Basic structure of the proposed thermostat. (**b**) Operation principle of the proposed thermostat.

The proposed circuit is similar to that in [5], and the basic structure is shown in Figure 3(a). To further reduce the chip area and release the number of bits, the two oscillators and corresponding time amplifiers were used to replace the linear delay lines and MUXs. A temperature-dependent delay circuit (TDDC) composed of an oscillator and a fixed-gain time amplifier (FGTA) was used to generate a thermal sensing delay *t_D_* PTAT. An adjustable-reference delay circuit (ARDC) composed of another oscillator with thermal compensation and an adjustable-gain time amplifier (AGTA) was used to program a temperature set-point delay *t_A_*. The characteristic curve on the thermal-compensated oscillator was designed to match that of the thermal oscillator to improve accuracy. The timing difference between *t_A_* and *t_D_* was detected by the time comparator to output *Comp/Locked*. As shown in Figure 3(b), the *Start* triggers the two oscillators and the corresponding period widths, t_d,osc_ and t_c,osc_, are generated. Then, the PTAT delay *t_D_* related to t_d,osc_ is produced after time amplification. In the meanwhile, with t_c,osc_ and the adjustable set-point values, the set-point delay *t_A_* is generated correspondingly. Finally, the states of *Comp* and *Locked* can be determined according to the relative delay between *t_A_* and *t_D_*. The following subsections provide detailed descriptions of the sub-circuits.

### 2.1. TDDC for Temperature Sensing

**Figure 4.**
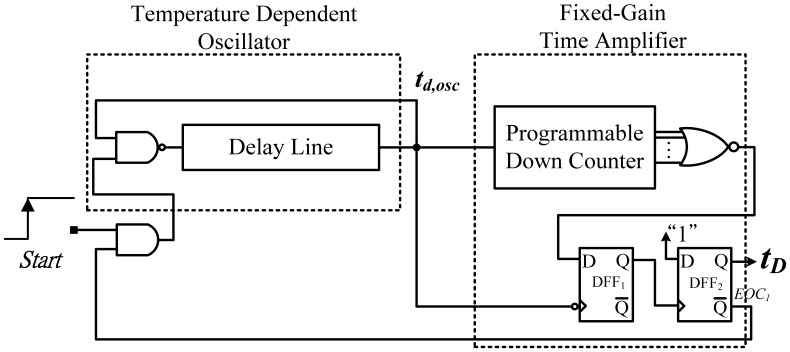
Block diagram of the TDDC.

Figure 4 shows the detailed circuit of the TDDC. For temperature sensing, a retriggerable ring oscillator was composed of a NAND gate and a buffer-based delay line. The oscillatory period t_d,osc_ was determined by the propagation delay of those logic gates. The period width has a linear relation to the temperature variation, and can be used to sense the temperature [5–9]. Consequently, the oscillator can act as a time-domain PTAT sensor, which is simpler than voltage-domain sensors. The simple sensor has inferior linearity because a curvature occurs on the transfer curve of t_d,osc_. The FGTA, modified from that in [9], is used for time amplification to obtain a sufficient temperature resolution. The additional DFF_1_ was inserted for deglitching. A larger preset input value results in higher output circulation times of the oscillatory period and a longer thermal delay *t_D_*.

A cyclic delay line instead of a linear delay line was used to release the unreasonable delay line length requirement used in [5]. With the help of the FGTA, the chip size can be reduced considerably using the oscillator, and a sufficiently fine resolution can be achieved. After each conversion of the TDDC, the end-of-conversion (EOC_1_) signal is generated to shut down the retriggerable oscillator to save power. In reality, the fixed input value can be released as variable value for process variation to ensure a satisfactory resolution or enough test temperature ranges.

### 2.2. ARDC for Programming Digital Set-Point

**Figure 5.**
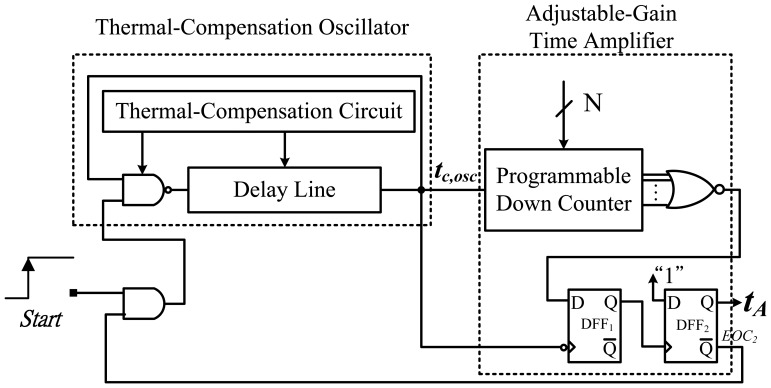
Block diagram of the ARDC.

As a replacement for numerous thermal-compensation delay cells and the corresponding delay adjustment MUX used in [5], the ARDC was designed for programming the temperature set-point delay *t_A_*, as shown in Figure 5. The AGTA can be regarded as a programmable timing generator with a digital control code or digital-to-time converter (DTC), similar to a DAC [10]. Its time resolution equals the oscillatory period width t_c,osc_. In the proposed thermostat, the timing generator does not use an elaborate circuit because the resolution is not expected to be fine, and the circuit design can be simplified. Conversely, for accuracy improvement, the period width of the oscillator must be sufficiently large to overcome the delay variation among various signal paths. The two amplifiers with identical bits were the same for delay matching, although the gain of FGTA was low. Consequently, the additional propagation delay induced by the amplifier can be counteracted to minimize the timing mismatch between *t_D_* and *t_A_*. The only difference is the fixed input value for time amplification and adjustable set-point value for diverse timing generation.

The thermal delay *t_D_* at the lower test temperature bound was usually larger than zero, and it caused an offset time. In [5], a lot of additional compensated delay cells were inserted at the beginning of the time reference delay line to compensate for this offset time. This resulted in a larger chip area and increased power consumption. In [6], the offset time cancellation circuit and designed trigger pulse width equal to the offset time were proposed to reduce the offset. However, this increased the circuit complexity. With the oscillator-based structure, the offset problem can be easily solved using one or two additional bit programmer down-counters. This does not considerably increase the design difficulty and chip area.

**Figure 6.**
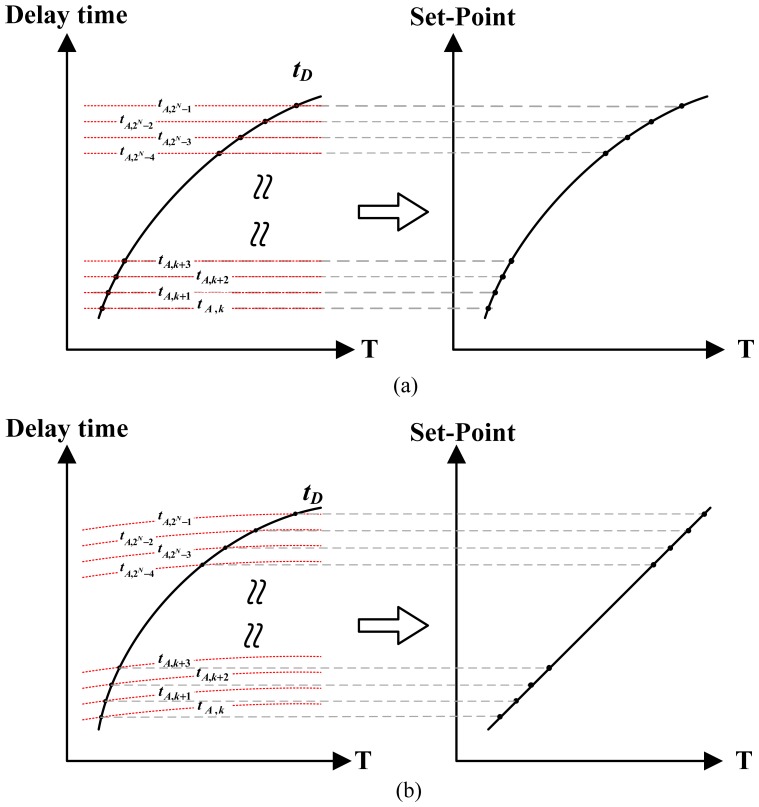
Linearity for (**a**) reference clock period and (**b**) curvature-compensating period.

Furthermore, the ARDC was based on a cyclic delay line structure that can further reduce the chip area because the numerous compensated delay cells and their thermal-compensation circuits can be removed. Compared to the thermal oscillator, the period width of t_c,osc_ must be temperature insensitive to act appropriately as a time reference. Although a reference clock can be easily used to replace t_c,osc_ for the reference, the transfer curve of t_d,osc_ has some curvature, and the thermostat accuracy is limited. A feasible linearization technique for lowering the nonlinearity effect on the accuracy is to compensate for the curvature of t_d,osc_ using that of t_c,osc_ [6], as shown in Figure 6. Therefore, the on-chip compensated oscillator rather than off-chip clock was used in this circuit for curvature compensation.

**Figure 7.**
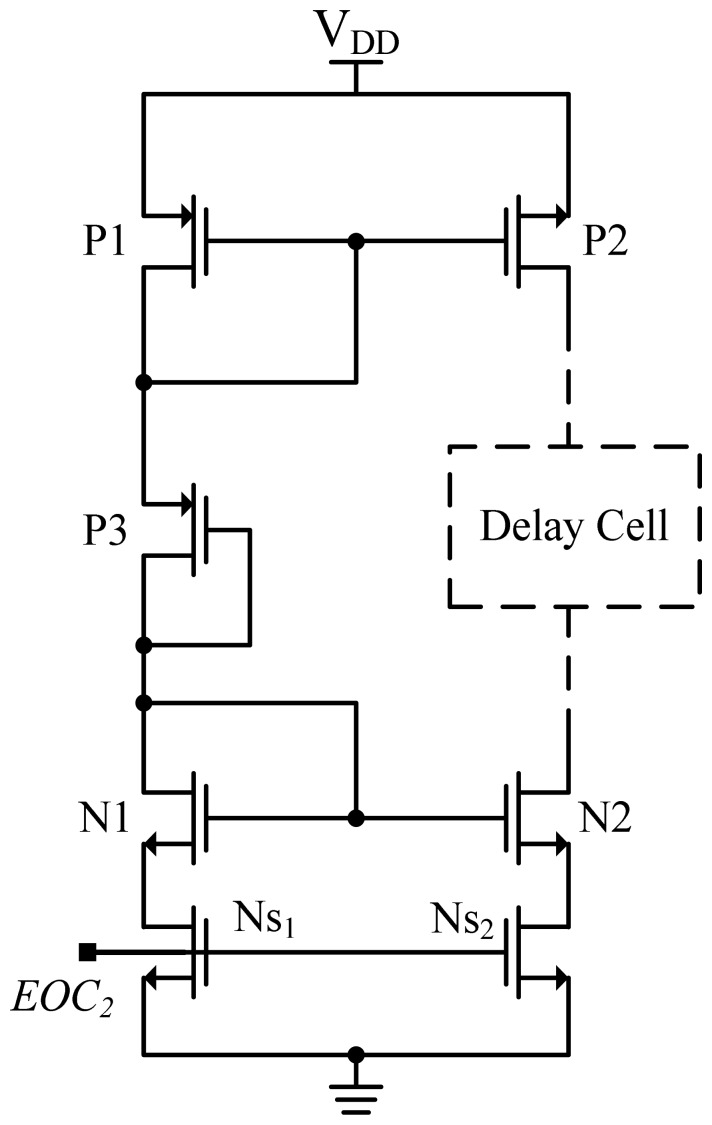
Schematic of thermal-compensation circuit.

The same thermal-compensation circuit also adopted in [6] was used to reduce the thermal-sensitivity of the NOT (or NAND) gates in the oscillator, and achieve curvature compensation, as shown in Figure 7. The diode-connected Transistors P1, N1 and P3 serve as the core of the thermal compensation circuit. Since P1, P3 and N1 are all diode connected, they will operate in saturation if bias current is flowing. The optimum bias voltage can be derived as [6]:

(1) $$V_{GS,P3}=V_{T}(T_{0})+\alpha (T-T_{0)})+2\frac{\alpha \cdot T}{km}$$

The sizes of transistors P1 and N1 are adjusted to make the gate to source voltage of P3 fit the requirement stated in Equation (1) as closely as possible and the corresponding conduction current of transistor P3 is shown to be:

(2) $$I_{D,P3}=\frac{1}{2}\mu_{0}C_{OX}\left(\frac{W}{L}\right){\left(\frac{T}{T_{0}}\right)}^{km}{\left[\frac{2\alpha T}{km}\right]}^{2}(1+\lambda V_{GS,P3})$$

When *km* equals to −2, the conduction current becomes totally temperature-independent. Although the actual value of *km* spreads over −1.2∼−2.0 [11], the temperature dependence of delay cell is still reduced greatly. Equations (1) and (2) can be provided the first cut design to generate the period width of the oscillator with low thermal sensitivity. Then, the size of the thermal-compensation Transistors P3, P1, and N1 must be properly adjusted to make the curvature of t_c,osc_ similar to that of t_d,osc_. The Ns_1_ switch, which is controlled by EOC_2_, was used to shut down the quiescent current of the thermal-compensation circuit to reduce power consumption. To effectively match the source resistances of Ns_2_ and Ns_1_, the Ns_2_ switch is added to reduce the current mirror error. Similarly, the operation of the compensated oscillator can be turned off using ECO_2_ to further reduce power consumption.

**Figure 8.**
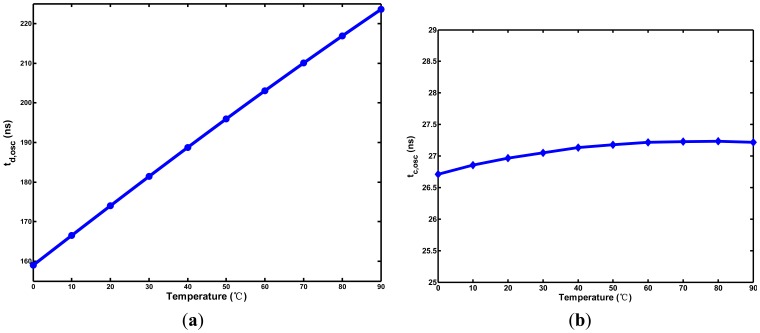
Simulated period widths for (**a**) t_d,osc_ and (**b**) t_c,osc_.

The simulation results of the two oscillators are shown in Figure 8. As descried above, the temperature-to-period transfer curve of t_d,osc_ with curvature is slightly convex. The curve of t_c,osc_ is similar to the one in Figure 6(b) for curvature compensation. To decrease the power dissipation, the operating frequencies of two oscillators are designed to be low.

### 2.3. Time Comparator with Built-in Hysteresis

**Figure 9.**
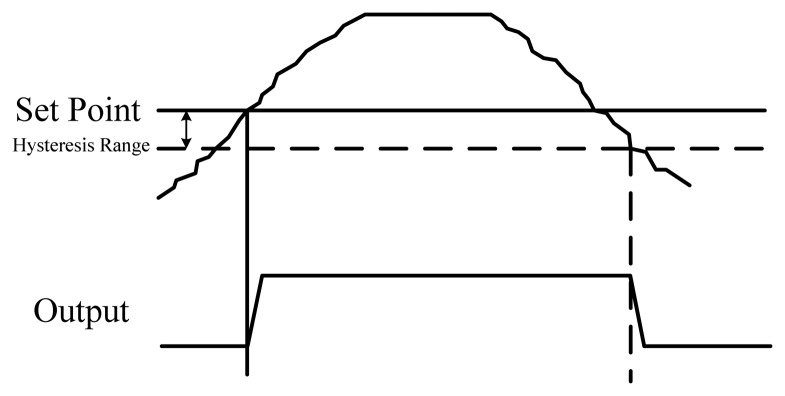
Hysteresis for chattering prevention.

The proposed circuit with a programmable digital set-point was designed for use in thermal management applications. To prevent output chattering when the measured temperature is at (or near) the programmed trip point values, the thermostat must have the hysteresis of the desired temperature range. Figure 9 shows that the output remains in the active state until the temperature falls under the additional setting range. This hysteresis also can provide the function of temperature control to maintain the temperature of the system near a desired set-point.

**Figure 10.**
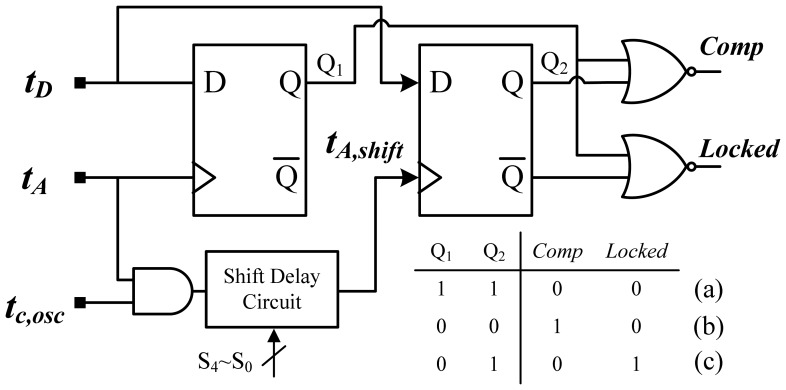
Schematic of time comparator.

**Figure 11.**
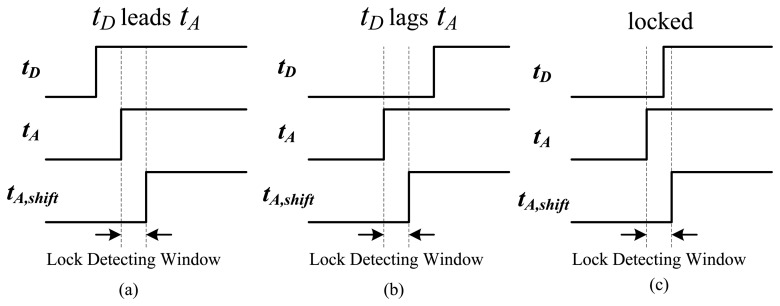
Operation principle with three differing states.

For over-temperature protection, the proposed time comparator with built-in custom hysteresis was used to determine the lead or lag relation between t_D_ and t_A_. The schematic of the comparator is plotted in Figure 10 and it was modified from the phase detector in [12]. The shift delay circuit realized with a 5-bit AGTA can form the hysteresis of the desired temperature range. Two DFFs were used to sample the input signals, and the shift delay circuit set up a detecting window, formed by t_A_ and t_A,shift_, to avoid chattering. The operation principle with three differing states is shown in Figure 11. In Figure 11(a), *t_D_* leads *t_A_* and *t_A,shift_*, which indicates that the test temperature does not exceed the set trip temperature. Consequently, an alarm signal cannot be activated. In Figure 11(b), the alarm is activated because the test temperature exceeds the set temperature. The output remains in the previous status when *t_D_* enters the detecting window, as shown in Figure 11(c). The same operation principle can also be used for under-temperature protection. The hysteresis of the temperature range can be set by the input value of the shift delay circuit and temperature resolution. For example, the 0.05 °C resolution and 5-bit design in the shift delay circuit can achieve a maximal hysteresis of approximately 1.6 °C. Because no hard limits occur in time-domain sensors, the time resolution t_c,osc_ in the ARDC can be easily expanded to avoid time comparison errors caused by a dead zone or sampling window of the DFF. This substantially reduces the complexity of the circuit design.

## 3. Measurement Results

**Figure 12.**
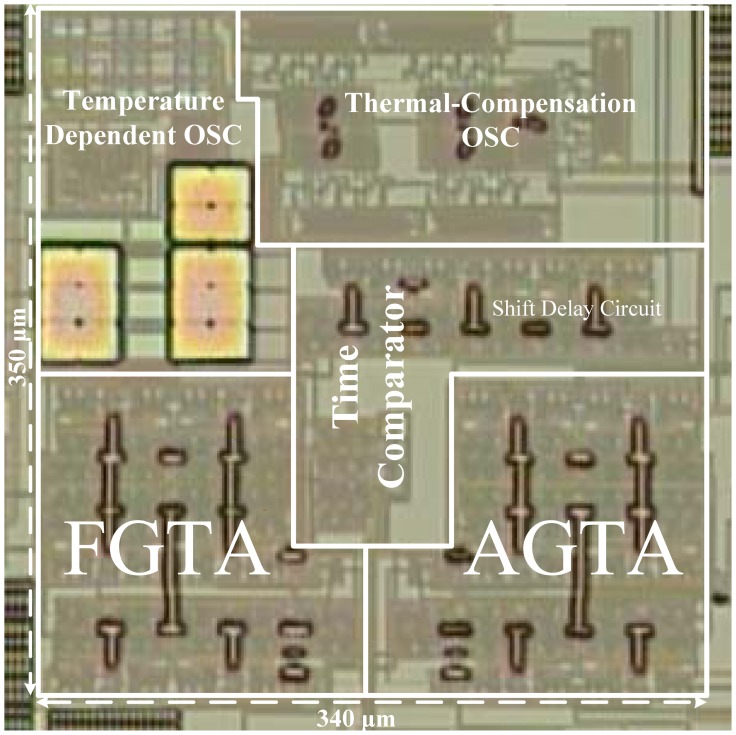
Microphotograph of the proposed circuit.

A microphotograph of the proposed thermostat fabricated with a TSMC 0.35-μm CMOS process is shown in Figure 12. With the oscillator-based structure, the chip area of 0.12 mm^2^ was less than that (0.4 mm^2^) of its delay-line-based predecessor [5]. This achieves more than three-fold improvement in chip size. The temperature resolution was improved without considerably increasing the chip area. In addition, to minimize the effect of process variation and element mismatch, both amplifiers were symmetrically laid out as close to each other as possible for delay matching. With a single supply voltage of 3.0 V, the power consumption was 30 μW at a sample rate of 10 samples/s.

**Figure 13.**
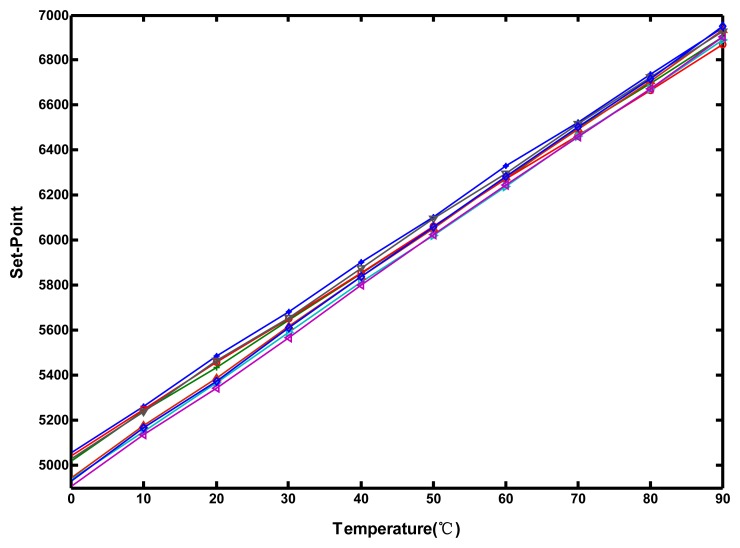
Trip temperature versus programmed set-point for eight test chips.

To determine the performance of the proposed circuit, measurements were performed in intervals 10 °C with a 0–90 °C temperature range in a programmable temperature and humidity chamber (MHG-120AF). The step input signal *Start* was issued using the FPGA control board. During the increase of test temperature, the input value of AGTA from low to high gradually was varied to evaluate the relationship between the trip temperature and the programmed set-point. The corresponding set-point value of the temperature at the time was determined according to the output of the time comparator. The measurement results for eight packaged chips are shown in Figure 13.

**Figure 14.**
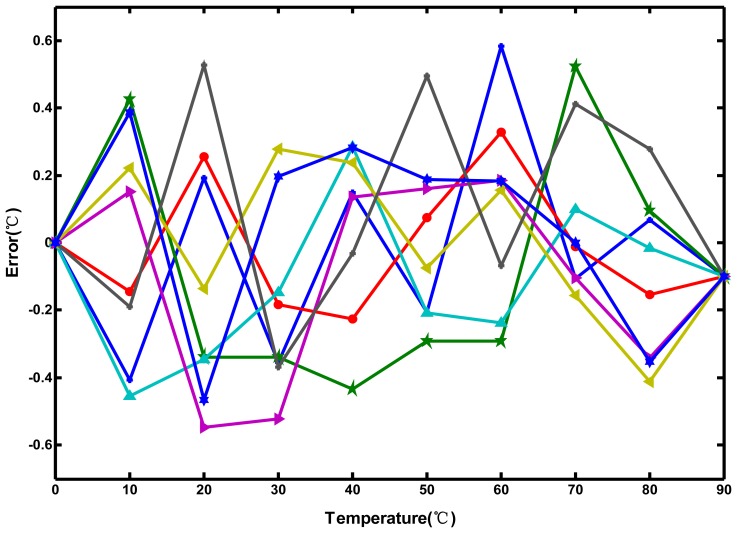
Measurement errors after two-point calibration for eight chips.

A simple straight calibration line for the measurement results of each individual chip was fulfilled off-line by performing linear curve fitting with the set-point values of 0 °C and 90 °C, which were chosen to minimize error. The achieved inaccuracy was within ±0.6 °C after two-point calibration, as shown in Figure 14. Although the same curvature compensation technique was used in the proposed circuit and [6], the error was larger than that (±0.3 °C) in [6], which used a linear delay-line-based structure. The oscillator-based structure may decrease the accuracy because of the use of two oscillators and more complex circuit operation. However, its error is sufficient for on-chip thermal monitoring. In other words, the technique resulted in a 1.5-fold accuracy improvement compared to the linear delay-line-based sensor [5].

**Figure 15.**
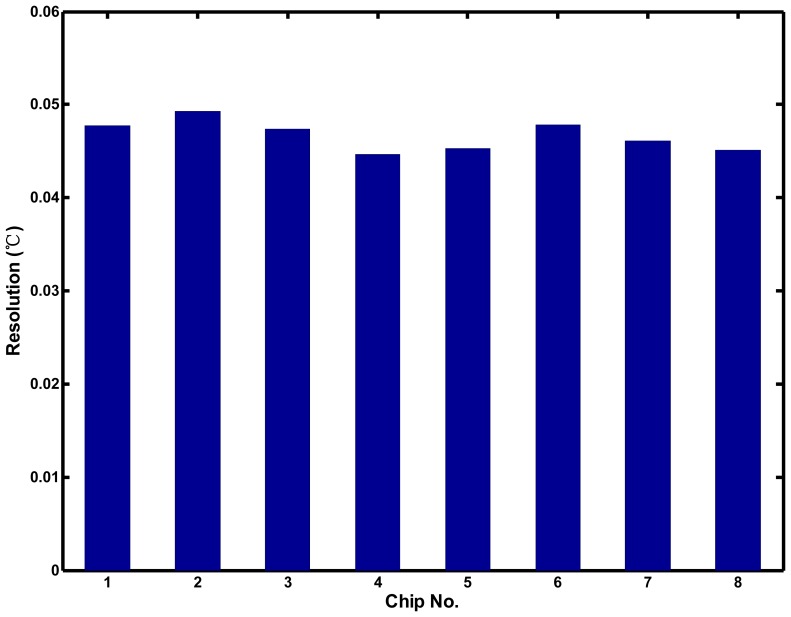
Effective resolution variation for eight chips.

**Figure 16.**
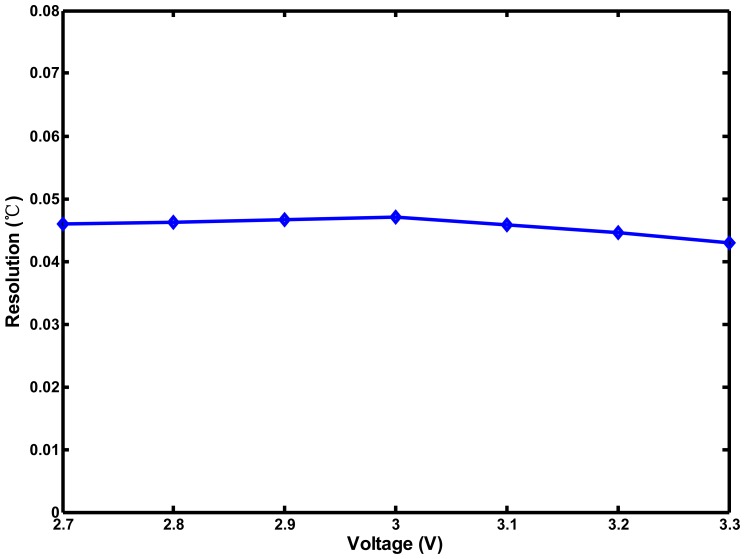
Effective resolution variation under ±10% supply voltage variation.

Figure 15 shows that the effective resolutions for eight chips were 0.045–0.049 °C, which are finer than those of most sensors [5–9]. The chip-to-chip variation of the resolution was only ±4%. Similarly, to demonstrate the supply voltage sensitivity of the proposed thermostat, the supply voltage was adjusted from 2.7 to 3.3 V in increments of 0.1 V. The generated delays of the TDDC and ARDC compensated for each other when the supply voltage varied, and the effective resolution of the proposed circuit was less sensitive to the supply voltage variation. The resolution varied from 0.043 to 0.047 °C or an equivalent ±4.5% variation, as shown in Figure 16. The proposed sensor owns good immunity to not only process but also voltage variations.

## 4. Conclusions

This paper presents a CMOS time-domain thermostat with digital set-point programming with a small chip area of 0.12 mm^2^ in a TSMC 0.35 μm CMOS digital process and a low power consumption of approximately 30 μW at a sample rate of 10 samples/s. Compared to conventional circuits, which require a DAC or external resistor, the time-domain circuit with an on-chip thermal-compensation oscillator and a timing generator was used to program the set-point temperature. A custom hysteresis of the temperature range in the time comparator can be easily adjusted by off-chip setting. The proposed circuit was designed to release unreasonable delay line length requirements by replacing the delay-line-based structure with an oscillator-based structure. In addition, the temperature resolution can be improved without substantially increasing the chip area. The thermostat achieved a resolution better than 0.05 °C and a ten-fold improvement in resolution. The time-domain curvature compensation technique was used for accuracy enhancement. With two-point calibration and a temperate range of approximately 0–90 °C, the measurement error was within ±0.6 °C, which is sufficient for on-chip thermal management systems. These specifications demonstrate the proposed circuit is very suitable for low-power and low-cost VLSI systems. The measured performances of the proposed thermostat and other time-domain temperature sensors are shown in Table 1 for comparison.

## Figures and Tables

**Table 1.** Measured performances among time-domain works for easy comparison.

**Sensor**	**Resolution (°C)**	**Range (°C)**	**Error (°C)**	**Power Consumption**	**Area (mm^2^)**	**CMOS Technology (μm)**
This Work	0.05	0∼90	±0.6	30 μW@10 Hz	0.12	0.35
[5]	0.5	0∼75	±1.0	9 μW@20 Hz	0.4	0.35
[6]	0.09	0∼90	±0.3	36.7 μW@2 Hz	0.6	0.35
[7]	0.3	0∼100	−1.6∼3	0.22 μW@100 Hz	0.05	0.18
[8]	0.78	0∼100	±4 ^#^	1.2 mW@5 kHz	0.12	0.13
[9]	0.133	0∼100	−0.7∼0.6 ^#^	175 μW@1 kHz	NA	0.18/0.22

# one-point calibration.
